# Anti-Fatigue Effect by Peptide Fraction from Protein Hydrolysate of Croceine Croaker (*Pseudosciaena crocea*) Swim Bladder through Inhibiting the Oxidative Reactions including DNA Damage

**DOI:** 10.3390/md14120221

**Published:** 2016-12-13

**Authors:** Yu-Qin Zhao, Li Zeng, Zui-Su Yang, Fang-Fang Huang, Guo-Fang Ding, Bin Wang

**Affiliations:** Zhejiang Provincial Engineering Technology Research Center of Marine Biomedical Products, School of Food and Pharmacy, Zhejiang Ocean University, 1st Haidanan Road, Changzhi Island, Lincheng, Zhoushan 316022, China; zhaoy@hotmail.com (Y.-Q.Z.); 9001000@163.com (L.Z.); yangzs87@163.com (Z.-S.Y.); gracegang@126.com (F.-F.H.)

**Keywords:** croceine croaker (*Pseudosciaena crocea*), swim bladder, peptide, antioxidant activity, anti-fatigue activity

## Abstract

The swim bladder of the croceine croaker (*Pseudosciaena crocea*) was believed to have good curative effects in various diseases, including amnesia, insomnia, dizziness, anepithymia, and weakness after giving birth, in traditional Chinese medicine. However, there is no research focusing on the antioxidant and anti-fatigue peptides from croceine croaker swim bladders at present. Therefore, the purpose of this study was to investigate the bioactivities of peptide fractions from the protein hydrolysate of croceine croaker related to antioxidant and anti-fatigue effects. In the study, swim bladder peptide fraction (SBP-III-3) was isolated from the protein hydrolysate of the croceine croaker, and its antioxidant and anti-fatigue activities were measured using in vitro and in vivo methods. The results indicated that SBP-III-3 exhibited good scavenging activities on hydroxyl radicals (HO•) (EC_50_ (the concentration where a sample caused a 50% decrease of the initial concentration of HO•) = 0.867 mg/mL), 2,2-diphenyl-1-picrylhydrazyl radicals (DPPH•) (EC_50_ = 0.895 mg/mL), superoxide anion radical ($O_{2}^{-}$•) (EC_50_ = 0.871 mg/mL), and 2,2′-azino-bis-3-ethylbenzothiazoline-6-sulfonic acid radical (ABTS^+^•) (EC_50_ = 0.346 mg/mL). SBP-III-3 also showed protective effects on DNA damage in a concentration-effect manner and prolonged the swimming time to exhaustion of Institute of Cancer Research (ICR) mice by 57.9%–107.5% greater than that of the control. SBP-III-3 could increase the levels of muscle glucose (9.4%–115.2% increase) and liver glycogen (35.7%–157.3%), and decrease the levels of blood urea nitrogen (BUN), lactic acid (LA), and malondialdehyde (MDA) by 16.4%–22.4%, 13.9%–20.1%, and 28.0%–53.6%, respectively. SBP-III-3 also enhanced the activity of lactic dehydrogenase to scavenge excessive LA for slowing the development of fatigue. In addition, SBP-III-3 increased the activities superoxide dismutase, catalase, and glutathione peroxidase to reduce the reactive oxygen species (ROS) damage in mice. In conclusion, SBP-III-3 possessed good anti-fatigue capacities on mice by inhibiting the oxidative reactions and provided an important basis for developing the swim bladder peptide functional food.

## 1. Introduction

Fatigue is one of the most common and disabling non-motor problems, which generally leads to negative effects on physical and cognitive function. Therefore, fatigue is best defined as the difficulty in initiating or sustaining voluntary activities, and classified into mental and physical fatigue [1]. Exercise-induced fatigue usually associates with increased stress levels caused by modern lifestyles, along with a decline in exercise performance [2]. At present, several theories including “exhaustion theory” and “radical theory” have been put forward to explain the mechanisms of exercise-induced fatigue [1]. Among them, the “exhaustion theory” speculates that energy sources, including glucose and liver glycogen, will be exhausted during exercise, which is accompanied by physical fatigue. In this theory, some studies indicated that post-exercise nutrition through the administration of proteins, saccharides, and amino acids can eliminate the accumulated harmful metabolites, repair the damage of organisms, and facilitate recovery from fatigue [3]. Compared with these nutrient substances, protein hydrolysates and peptides have been widely studied due to their potential health benefits associated with high bioactivities, low molecular weight (MW), easy absorption, and less toxicity [4,5]. Ding et al. reported that jellyfish collagen hydrolysate could promote climbing endurance and had anti-fatigue effects in rats [2]. Wang et al. reported that the spleen-derived peptide CMS001 had anti-fatigue effects in mice. Therefore, bioactive protein hydrolysates and peptides are believed to be helpful for counteracting and ameliorating physical fatigue [6].

Except for exhaustion of energy sources, high-intensity exercise often destroys the balance between the oxidation system and anti-oxidation system of human body. The accumulated reactive oxygen species (ROS) will put the body in a state of oxidative stress and bring injury to the body by attacking biomacromolecules and cell organs [6,7]. Some reports found that exogenous dietary antioxidants can decrease the contribution of exercise-induced oxidative stress and improve the animal’s physiological condition [5]. You et al. reported that loach peptide could scavenge hydroxyl radical (HO•) and, 2,2-diphenyl-1-picrylhydrazyl radical (DPPH•) in vitro and increase the activities of superoxide dismutase (SOD), catalase (CAT), and glutathione peroxidase (GSH-Px) in vivo [1]. Wei et al. reported that high Fischer ratio oligopeptides derived from food sources such as corn, tuna, and *pinctada martensii*, could scavenge free radicals in vitro and increase the activities of SOD, CAT, and GSH-Px in vivo [8]. In addition, high Fischer ratio oligopeptides could prolong the swimming time, increase liver glucogen contents, and lower blood urea nitrogen (BUN) and lactic acid (LA) levels of exercised mice. However, the anti-fatigue and anti-oxidation mechanisms of protein hydrolysates and peptides have not been fully elucidated. Therefore, more detailed research should be done seeking more high-efficiency antioxidant peptides used in the daily diet to reduce oxidative damage and fight against fatigue.

At present, large quantities of byproducts, accounting for 50%–70% of the original raw material, are generated during the aquatic products processing, and optimal use of these byproducts are an effective approach to protect the environment and produce value-added products to increase the revenue of fish processors [9]. Therefore, preparation of protein hydrolysates and peptides from fish byproducts are extensively researched [5,10]. Proteins in food resources possess a variety of active peptide sequences, and enzymatic hydrolysis is thought as an effective method to release those active fragments without destroying their nutritional value and adding hazardous substances, including residual organic solvents and toxic chemicals in the final products [4,5]. HO• attacks almost every molecule in living cells and is demonstrated to be a highly damaging species in free radical pathology. Thus, the removal of HO• is probably one of the most effective defenses against various diseases for a living body [10]. In our previous research, three antioxidant peptides including Tyr–Leu–Ser–Met–Ser–Arg (YLSMSR), Val–Leu–Tyr–Glu–Glu (VLYEE), and Met–Ile–Leu–Met–Arg (MILMR) were isolated from proteins hydrolysate of croceine croaker (*Pseudosciaena crocea*) muscle and showed strong DPPH•, HO•, superoxide anion radical ($O_{2}^{-}$•), and 2,2′-azino-bis-3-ethylbenzothiazoline-6-sulfonic acid radical (ABTS^+^•) scavenging activities [11]. Acid and pepsin-soluble collagens from croceine croaker scales were prepared and characterized [12], and antioxidant peptides including Gly–Phe–Arg–Gly–Thr–Ile–Gly–Leu–Val–Gly (GFRGTIGLVG), Gly–Pro–Ala–Gly–Pro–Ala–Gly (GPAGPAG), and Gly–Phe–Pro–Ser–Gly (GFPSG) from the acid-soluble collagen showed strong DPPH•, HO•, ABTS^+^•, and $O_{2}^{-}$• scavenging activities [13]. Traditional Chinese medicine considers that swim bladder of the croceine croaker to have good curative effects in various diseases, including amnesia, insomnia, dizzy, anepithymia, and weakness after giving a birth, and present researchers also suggest that it could serve to remove free radicals and ward against inflammation and cancer [14]. However, there was no research focusing on the antioxidant and anti-fatigue peptides from croceine croaker swim bladders. Therefore, the objectives of the present study were to prepare the active peptide fraction from croceine croaker swim bladders, and its bioactivities related to antioxidant and anti-fatigue effects of prepared fraction were also evaluated.

## 2. Results and Discussion

### 2.1. Preparation of Protein Hydrolysates of Swim Bladder and Their HO• Scavenging Activities

In the experiment, four proteases, including alcalase, papain, pepsin, and trypsin were used to hydrolyze the proteins of croceine croaker swim bladders, respectively. The degree of hydrolysis (DH) and the HO• scavenging activities were used to screen the most suitable enzymes for subsequent experiments, and HO• scavenging activities was expressed as EC_50_ (the concentration where a sample caused a 50% decrease of the initial concentration of HO•) (Table 1). The DH (%) of alcalase hydrolysate was 22.32% ± 0.74%, which was significantly higher than those of papain hydrolysate (17.84% ± 0.71%), pepsin hydrolysate (19.52% ± 0.49%), trypsin hydrolysate (16.21% ± 0.37%), and neutrase hydrolysate (21.37% ± 0.67%) (*p* < 0.05). The EC_50_ value of alcalase hydrolysate was 8.85 mg/mL, which was significantly lower than those of papain hydrolysate (11.76 mg/mL), pepsin hydrolysate (13.68 mg/mL), and trypsin hydrolysate (10.02 mg/mL) (*p* < 0.05). The result was in accordance with the previous reports that high DH and low MW of hydrolysates made a great contribute to their antioxidant activities including HO• scavenging activity [15,16]. Proteases digest long protein chains into shorter fragments by splitting the peptide bonds that link amino acid residues. Due to the specificity of enzymes reactions, protein hydrolysates from the same proteins hydrolyzed using different proteases exhibit different DH and bioactivities because the obtained peptides are diverse in terms of chain length and amino acid sequence [5]. Therefore, alcalase hydrolysate (designated as SBP) was selected for further study.

### 2.2. Preparation of Antioxidant Peptides from SBP

#### 2.2.1. Fractionation of SBP by Ultrafiltration

Protein hydrolysate is composed of peptides with different molecular sizes and free amino acids, and their bioactivities are influenced by the molecular size of peptides. Therefore, ultrafiltration is a popular method for fractionation and enrichment concentration of peptides with specific molecular sizes from hydrolysates [17]. To obtain purified swim bladder peptide, SBP was fractionated by ultrafiltration with three molecular weight (MW) cut-off membranes of 10, 5, and 3 kDa in turn, respectively, and the resulting four fractions were prepared and named as SBP-I (MW < 3 kDa), SBP-II (3 kDa < MW < 5 kDa), SBP-III (5 kDa < MW < 10 kDa),, and SBP-IV (MW > 10 kDa), respectively. The yields of SBP-I, SBP-II, SBP-III, and SBP-IV were 10.08, 17.30, 23.37, and 28.97 mg protein/g swim bladder, respectively.

For acquiring the fraction with high antioxidant activity, HO• scavenging activities of four prepared fractions were measured, and the results indicated that HO• scavenging activities of SBP, SBP-I, SBP-II, SBP-III, and SBP-IV were 50.32% ± 0.90%, 34.89% ± 2.01%, 40.63% ± 1.59%, 58.36% ± 1.72%, and 52.57% ± 2.20%, respectively, at the concentration of 5 mg/mL. SBP-III showed significantly higher antioxidant activity than SBP and the other three fractions at the tested concentrations (*p* < 0.05). In addition, the EC_50_ of SBP-III was 3.579 mg/mL and significantly less than that of SBP (8.85 mg/mL). The result was in line with the previous report that that samples with lower average molecular weights possibly contained more substrates, which were electron donors and could react with free radicals to convert them to more stable products and terminate the radical chain reactions [9]. From the data, it could be concluded that SBP-III contained more effective antioxidant peptides and could be chosen for subsequent separation.

#### 2.2.2. Gel Filtration Chromatography of SBP-III

**Figure 1:**
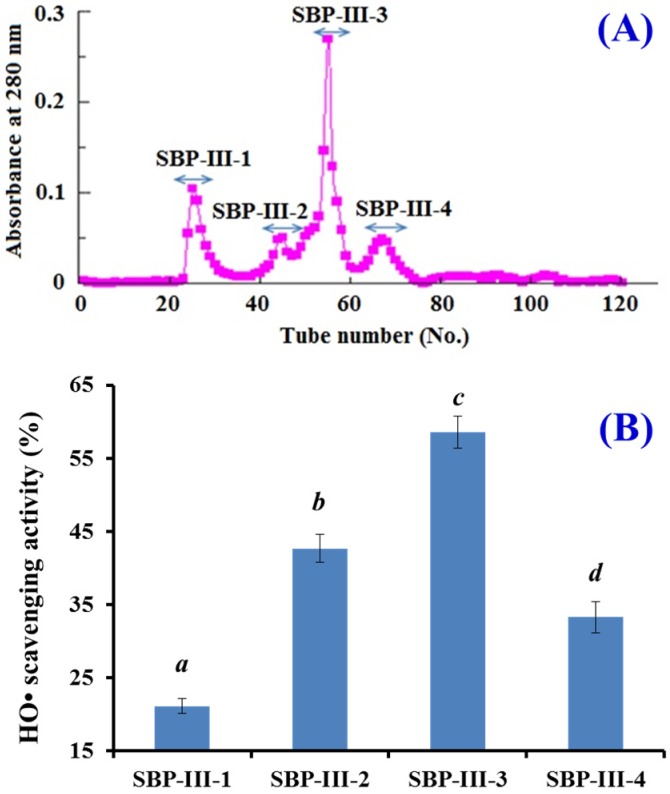
(**A**) Gel filtration chromatography of SBP-III on a Sephadex G-25 column; and (**B**) HO• scavenging activities of subfractions from SBP-III. All of the values were mean ± SD (*n* = 3). *^a^*^–*d*^ Columnwise values with the same superscripts of this type indicated no significant difference (*p* > 0.05).

Gel filtration chromatography is an effective separation technique on the basis of molecule size and widely applied to separate components in a mixture [10]. As shown in Figure 1, SBP-III was separated into four subfractions (SBP-III-1 to SBP-III-4) using a Sephadex G-25 column. From the linear equation (Log MW = −0.2036R_t_ + 7.6164, *R*^2^ = 0.9766), the MWs of four subfractions were 9.48 kDa (SBP-III-1), 7.74 kDa (SBP-III-2), 6.78 kDa (SBP-III-3), and 5.79 kDa (SBP-III-4), respectively. HO• scavenging activities of SBP-III-3 was 58.53% ± 2.17% at the concentration of 1 mg/mL, which was significantly higher than those of SBP-III-1 (21.15% ± 1.03%), SBP-III-2 (42.67% ± 1.94%), and SBP-III-4 (33.27% ± 2.11%), respectively (*p* < 0.05). The data indicated that SBP-III-3 could effectively restrain the production of HO• and terminate the radical chain reaction. Pan et al. reported that the hydrolysate subfraction from the skate (*Raja porosa*) cartilage protein using a Sephadex G-15 column had higher radical scavenging activity than other subfractions with smaller molecular size [10]. These results indicated that some factors, including amino acid composition and sequence, may also influence the activities of peptides in addition to the MW. Therefore, SBP-III-3 was chosen for further evaluation on in vitro antioxidant activity and the in vivo anti-fatigue effect.

### 2.3. In Vitro Antioxidant Activity of SBP-III-3

#### 2.3.1. HO• Scavenging Activity

**Figure 2:**
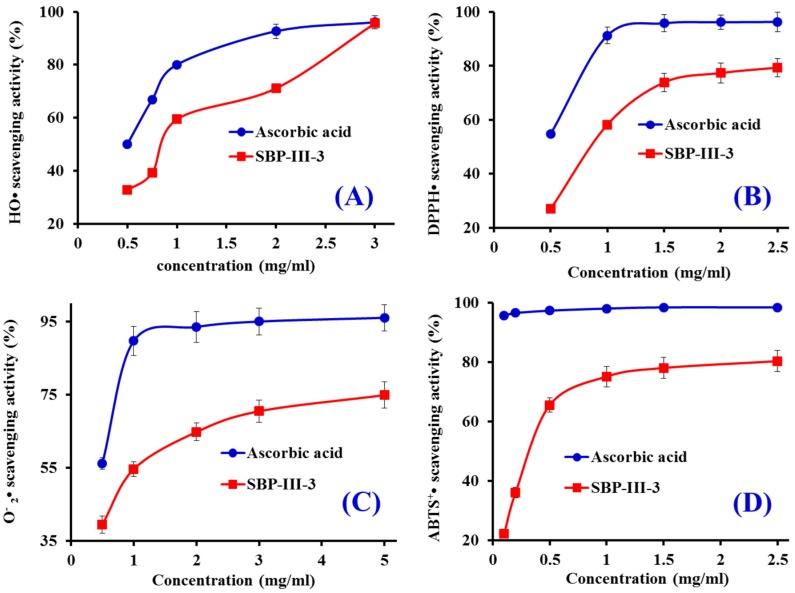
HO• (**A**), DPPH• (**B**), $O_{2}^{-}$• (**C**), and ABTS^+^• (**D**) scavenging activities of SBP-III-3. All of the values were mean ± SD (*n* = 3).

HO• is a highly reactive radical to the organism because it can destroy virtually all types of macromolecules including carbohydrates, nucleic acids (mutations), lipids (lipid peroxidation), and amino acids (e.g., conversion of Phe to m-Tyrosine and o-Tyrosine). As shown in Figure 2A, the HO• scavenging rate of SBP-III-3 showed a dose-response relationship, and the EC_50_ of SBP-III-3 was 0.867 mg/mL, which was lower than those of Pro–Ser–Tyr–Val (PSYV) (2.64 mg/mL) [18], Pro–Ser–Lys–Tyr–Glu–Pro–Phe–Val (PSKYEPFV) (2.86 mg/mL) [19], Pro–Tyr–Ser–Phe–Lys (PYSFK) (2.283 mg/mL), Gly–Phe–Gly–Pro–Leu (GFGPL) (1.612 mg/mL), Val–Gly–Gly–Arg–Pro (VGGRP) (2.055 mg/mL) [20], Phe–Ile–Met–Gly–Pro–Tyr (FIMGPY) (3.037 mg/mL), Gly–Pro–Ala–Gly–Asp–Tyr (GPAGDY) (3.92 mg/mL), and Ile–Val–Ala–Gly–Pro–Gln (IVAGPQ) (5.03 mg/mL) [10] from protein hydrolysates of weatherfish loach muscle, grass carp muscle and skin, and skate cartilages. SBP-III-3 showed good HO• scavenging activity, which demonstrated that it could serve as a scavenger for reducing the damage induced by HO• in biological systems.

#### 2.3.2. DPPH• Scavenging Activity

DPPH• scavenging assay is popular and efficient in predicting the antioxidant activities of protein hydrolysates and peptides. It has a deep violet colour in solution and generates a strong absorption band at about 517 nm. The solution becomes colourless or pale yellow following the reduction of the absorption value at 517 nm when the radicals are neutralized [21]. Therefore, DPPH• scavenging activity of SBP-III-3 was measured and shown in Figure 2B. SBP-III-3 scavenged DPPH• in a concentration-effect manner with EC_50_ of 0.895 mg/mL, but its activity was lower than the positive control of ascorbic acid. In addition, the EC_50_ of SBP-III-3 was lower than those of PSYV (17.0 mg/mL) [18], Phe–Leu–Asn–Glu–Phe–Leu–His–Val (FLNEFLHV) (4.950 mg/mL) [22], Thr–Thr–Ala–Asn–Ile–Glu–Asp–Arg–Arg (TTANIEDRR) (2.503 mg/mL) [23], FIMGPY (2.60 mg/mL), GPAGDY (3.48 mg/mL) and IVAGPQ (3.93 mg/mL) [10], PYSFK (1.575 mg/mL) [20], and Leu–Leu–Pro–Phe (LLPF) (1.084 mg/mL) [24] from hydrolysates of loach, blue mussel, bluefin leatherjacket, salmon pectoral fin, skate cartilage, grass carp skin, and corn gluten meal, but it was higher than those of Gly–Ser–Gln (GSQ) (0.61 mg/mL) [25], Pro–Ile–Ile–Val–Tyr–Trp–Lys (PIIVYWK) (0.713 mg/mL) [20], His–Phe–Gly–Asp–Pro–Phe–His (HFGBPFH) (0.20 mg/mL) [26], Phe–Leu–Pro–Phe (FLPF) (0.789 mg/mL), and Leu–Pro–Phe (LPF) (0.777 mg/mL) [24] from protein hydrolysates of Chinese leek, blue mussel, grass carp skin, mussel sauce and corn gluten meal. Therefore, these results indicated that SBP-III-3 had the strong ability to donate an electron or hydrogen radical for inhibiting the DPPH• reaction.

#### 2.3.3. $O_{2}^{-}$• Scavenging Activity

$O_{2}^{-}$• can promote oxidative reaction to generate H_2_O_2_ and HO• to damage the biomacromolecule because it can release protein-bound metals and form perhydroxyl radicals which initiate lipid oxidation. The $O_{2}^{-}$• scavenging activity of SBP-III-3 was increased with increasing concentration ranged from 0.5 mg/mL to 5.0 mg/mL (Figure 2C). The IC_50_ value of SBP-III-3 was 0.871 mg/mL, which was lower than those of MILMR (0.993 mg/mL) [11], FIMGPY (1.61 mg/mL), GPAGDY (1.66 mg/mL), and IVAGPQ (1.82 mg/mL) [10] from protein hydrolysates of croceine croaker muscle and skate cartilage. Therefore, SBP-III-3 might have ability to remove $O_{2}^{-}$• damage in biological systems.

#### 2.3.4. ABTS^+^• Scavenging Activity

The blue/green ABTS^+^• produced by oxidation of ABTS with K_2_S_2_O_8_ has an absorption maximum of 734 nm and can be converted back to its colorless neutral form by antioxidants following the decrease of the absorption. As shown in Figure 2D, SBP-III-3 showed strong ABTS^+^• scavenging activity in a dose-effect manner with EC_50_ value of 0.346 mg/mL, which was lower than those of FLNEFLHV (1.548 mg/mL) [22], FLPF (1.497 mg/mL), LPF (1.013 mg/mL), LLPF (1.031 mg/mL) [24], FIMGPY (1.04 mg/mL), GPAGDY (0.77 mg/mL), and IVAGPQ (1.29 mg/mL) [10], VGGRP (0.465 mg/mL) [20], Trp–Glu–Gly–Pro–Lys (WEGPK) (5.407 mg/mL), Gly–Pro–Pro (GPP) (2.472 mg/mL), and Gly–Val–Pro–Leu–Thr (GVPLT) (3.124 mg/mL) [17] from protein hydrolysates of salmon, corn gluten meal, skate cartilage, grass carp skin, and bluefin leatherjacket heads. These results indicated that SBP-III-3 could convert ABTS^+^• to its colorless neutral form and block the free radical reaction.

#### 2.3.5. Protective Activity against Free Radical-Induced DNA Damage

**Figure 3:**
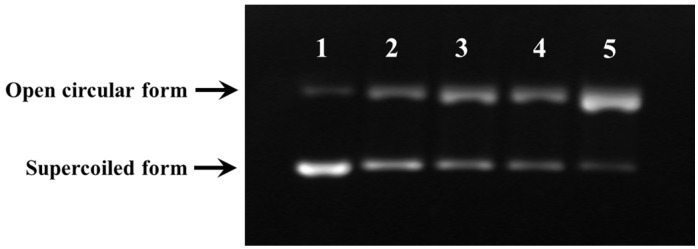
DNA damage protective effect of SBP-III-3. Lane 1, the native pBR322DNA; lanes 2, the DNA treated with FeSO_4_, H_2_O_2_ and SBP-III-3 (3.0 mg/mL); lane 3, the DNA treated with FeSO_4_, H_2_O_2_, and ascorbic acid (1.0 mg/mL); lane 4, the DNA treated with FeSO_4_, H_2_O_2_, and SBP-III-3 (1.0 mg/mL); lane 5, the pBR322DNA treated with FeSO_4_ and H_2_O_2_.

In the organism, the excessive production of ROS may cause a quantity of degenerative processes such as cancer, premature aging, and cardiovascular and neurodegenerative diseases, while DNA damage is a key step in these ROS-induced effects [13,27]. Therefore, the protective activity of SBP-III-3 against oxidative damage of DNA induced by H_2_O_2_ was also evaluated and showed in Figure 3. The damage of plasmid DNA results in a cleavage of one of the phosphodiester chains and produces a relaxed open circular form. Further cleavage near the first breakage leads to linear double stranded DNA molecules. The formation of circular form of DNA is indicative of single-strand breaks and the formation of linear form of DNA is indicative of double-strand breaks [28]. The plasmid DNA (pBR322DNA) was mainly of the supercoiled form in the absence of FeSO_4_ and H_2_O_2_ (Figure 3, lane 1, control). HO• would be generated from iron-mediated decomposition of H_2_O_2_ when FeSO_4_ and H_2_O_2_ were added into the sample, and it subsequently broke the supercoiled DNA and converted the supercoiled form into the open circular form (Figure 3, lane 5). In the experiment, the linear form of DNA was not observed, which indicated that the generated HO• from iron-mediated decomposition of H_2_O_2_ might be too small and could not break the double-strand of DNA. As shown in Figure 3 (lanes 2, 3, and 4), the contents of supercoiled form of DNA was obvious higher than that of Figure 3 (lane 5). In addition, the contents of supercoiled form of DNA in Figure 3 (lane 2) were higher than that of Figure 3 (lane 4). These data indicated that both SBP-III-3 and the positive control of ascorbic acid could have protective effects on DNA damage in a concentration-effect manner. Therefore, SBP-III-3 could prevent the reaction of Fe^2+^ with H_2_O_2_ and directly scavenge HO• by donating a hydrogen atom or electron and, therefore, protecting the supercoiled plasmid DNA from HO• dependent strand breaks. This finding was in line with the result that SBP-III-3 could effectively scavenge HO• in HO• scavenging assay in vitro.

### 2.4. In Vivo Anti-Fatigue Effects of SBP-III-3

#### 2.4.1. SBP-III-3 Prolonged Exhaustive Swimming Time

**Figure 4:**
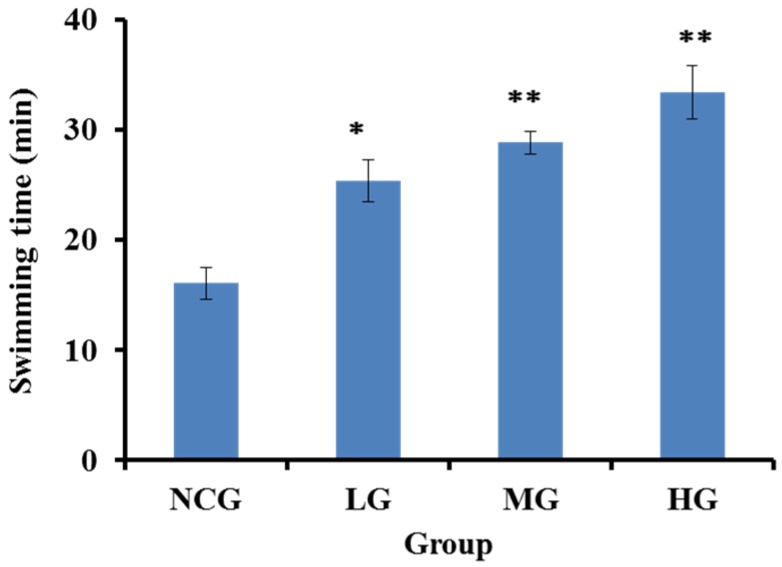
Effects of SBP-III-3 on loaded swimming time of mice. All the values were mean ± SD (*n* = 12). * *p* < 0.05, ** *p* < 0.01 compared with NCG.

Exercise tolerance assay is the most direct and objective indicators of reflecting physical fatigue. Swimming to exhaustion is an experimental exercise model to evaluate anti-fatigue; it works well for evaluating the endurance capacity of mice, and gives a high reproducibility [29]. The improvement of exercise endurance was the most powerful representation of anti-fatigue effect. In the experiment, the anti-fatigue effect of SBP-III-3 was investigated through the weight-loaded swimming test, and the length of the swimming time to exhaustion indicated the degree of fatigue. As shown in Figure 4, the mean exhaustion time of the SBP-III-3-HG was 33.41 ± 2.40 min (107.5% greater than that of NCG); the mean exhaustion time of the SBP-III-3-MG was 28.86 ± 1.01 min (79.2% greater than that of NCG); and the mean exhaustion time of the SBP-III-3-LG was 25.43 ± 1.91 min (57.9% greater than that of NCG) (*p* < 0.05 or *p* < 0.01). Therefore, the average loaded swimming time of mice was significantly longer in the SBP-III-3 treatment group (SBP-III-3-LG, SBP-III-3-MG, SBP-III-3-HG) than that of the normal control group (NCG) (16.1 ± 1.46 min) (*p* < 0.05 or *p* < 0.01), and these results indicate that SBP-III-3 has significant effects on movement and endurance in mice, thereby postponing the fatigue.

#### 2.4.2. Biologic Parameters Determination

##### SBP-III-3 Decreased Blood Urea Nitrogen (BUN)

Urea is formed in the liver as the end product of protein metabolism. During digestion, protein is broken down into small peptides and amino acids. The amino acid nitrogen is removed as NHt4, while the rest of the molecule is used to produce energy or other substances needed by the cell [30]. Thus, BUN is the metabolic outcome of amino acids and protein, and is one of the sensitive parameters related to fatigue. Therefore, it is usually applied to evaluate the tolerance capability when an animal suffers from a weight load. In other words, the less an animal is adapted to exercise, the more the BUN level increases [1]. As shown in Table 2, the BUN levels of the mice were significantly lower by 16.38%, 16.59%, and 22.06% in the SBP-III-3-LH, SBP-III-3-MG and SBP-III-3-HG compared to the NCG (*p* < 0.05 or *p* < 0.01), respectively. It was clear that SBP-III-3 treatment weakened the increase in BUN levels induced by catabolism of amino acids and proteins, which indicated that SBP-III-3 could reduce decomposition of proteins for energy, enhance adaptive capacity to physical load, and eventually improve tolerance capacity. The reduced protein metabolism of SBP-III-3 treatment groups is indicative of enhanced endurance.

##### SBP-III-3 Decreased Lactic Acid (LA)

The response to exercise in mammals begins with an increase in aerobic muscular activity, which switches over to anaerobic metabolism if the exercise is intense, which leads to the accumulation of LA [31]. Thus, the accumulation of blood serum LA is an important cause of fatigue. The increased content of LA will lower the pH in muscle tissue and blood, and induce some side effects of various physiological and biochemical processes, which affect both the cardio-circulating system and the skeletal muscle system function, and then do harm to the body performance [6]. The decrease in the contractive strength of the muscle eventually induces fatigue [32]. Therefore, LA was measured as another index to evaluate the level of fatigue. Table 2 showed the LA levels of the mice were significantly lower by 13.96%, 16.88%, and 20.13% in the SBP-III-3-LH, SBP-III-3-MG, and SBP-III-3-HG compared to the NCG (*p* < 0.05), respectively. The LA values of mice from these three groups had a similar trend in the BUN levels. The results indicated that reducing the LA levels might be an anti-fatigue pathway of SBP-III-3.

##### SBP-III-3 Increased the Activity of Lactic Dehydrogenase (LDH)

Serum LDH is known to be an accurate indicator of muscle damage, the normal function of LDH in cells is to catalyse the interconversion of pyruvate and lactate, thereby reducing the accumulation of LA in muscle [30]. As shown in Table 3, LDH activity was significantly higher in the SBP-III-3-MG (3605.87 ± 315.21 U/gprot) and SBP-III-3-HG (3690.76 ± 337.18U/gprot) compared to the NCG (2784.95 ± 322.92 U/gprot) (*p* < 0.05). LDH activity in the SBP-III-3-LG (3397.10 ± 215.90 U/gprot) was higher than that of the NCG (2784.95 ± 322.92 U/gprot), but the LDH activity showed no significant difference between SBP-III-3-LG and the NCG (*p* < 0.05). The present results suggested that SBP-III-3, especially high-dose groups could scavenge excessive LA by enhancing the activity of LDH, thereby slowing the development of fatigue.

##### SBP-III-3 Increased Liver and Muscle Glycogens

The level of energy stored as glycogen is of great importance in evaluating the capacity for high-intense exercise. Energy providing for exercise is derived initially from the decomposition of glycogen, and then from circulation glycogen released by the muscle and liver [33]. When the muscle glycogen level decreases during exercise, the reduction of liver glycogen may be the limiting factor in the capacity of endurance exercise. Thus, increasing the liver and muscle glycogen storage contributes to elevating the tolerance capacity and athletic capacity [33]. Fatigue will happen when the liver and muscle glycogen is mostly consumed [32]. Table 2 showed the liver glycogen levels of the mice in SBP-III-3-LH, SBP-III-3-MG and SBP-III-3-HG groups were increased by 35.70%, 108.65%, and 157.33%, respectively, and the liver glycogen levels in three treated groups were significantly higher than that in the NCG (*p* < 0.05 or *p* < 0.01). Muscle glycogen levels of the mice were significantly higher in the SBP-III-3-MG (3.39 ± 0.35 mg/g) and SBP-III-3-HG (4.80 ± 1.12 mg/g) compared to the NCG (2.23 ± 0.56mg/g) (*p* < 0.05). Muscle glycogen levels of the mice in the SBP-III-3-LG (2.44 ± 0.36 mg/g) were increased by 9.42% compared to the NCG, but the muscle glycogen level was no significant difference between SBP-III-3-LG and the NCG (*p* < 0.05). These results show that the anti-fatigue activity of SBP-III-3 may be related to the improvement in the metabolic control of exercise and the activation of energy metabolism [34].

#### 2.4.3. SBP-III-3 Enhanced the Antioxidant Enzymes and Decreased the Malondialdehyde (MDA)

Growing evidence indicates that reactive oxygen species (ROS) are responsible for exercise-induced protein oxidation, and contribute strongly to muscle fatigue [35]. ROS, including free radicals such as peroxyl radicals (ROO•), hydroxyl radicals (HO•), nitric oxide radicals (NO•), and superoxide radicals ($O_{2}^{-}$•), are physiological metabolites formed during aerobic life as a result of the metabolism of oxygen [5]. Under normal conditions, ROS are effectively eliminated by antioxidant defense systems, such as antioxidant enzymes and non-enzymatic factors. However, the balance between the generation and elimination of ROS is broken under pathological conditions, as a result of these events; bio-macromolecules are damaged by ROS-induced oxidative stress [36]. Muscle cells contain complex endogenous cellular defense mechanisms to clear up ROS to protect the body from exercise-induced oxidative injuries and DNA damages. For example: GSH-Px accelerates the reaction between H_2_O_2_ and glutathione (GSH) and converts them into H_2_O and oxidized GSH, SOD scavenges the $O_{2}^{-}$•, and CAT decomposes the HO•. Thus, the improvement in the activities of these defense mechanisms can help to fight against fatigue. Therefore, the present study investigated the activity of GSH-Px, SOD, and CAT to evaluate the anti-fatigue effects of SBP-III-3 on mice. As shown in Table 2, the SOD activities of SBP-III-3-HG and SBP-III-3-MG significantly increased by 44.2% and 15.7%, respectively, compared to the NCG (*p* < 0.05), while the SOD activity of SBP-III-3-LG increased by 4.2% and was without statistical significance compared to that of the NCG (*p* > 0.05). Moreover, the SOD activities between SBP-III-3-HG and SBP-MG showed a statistical difference (*p* < 0.01). The activities of GSH-Px and CAT showed a similar trend in the SOD activities. The GSH-Px activities of mice treated with SBP-III-3-HG, SBP-III-3-MG, and SBP-III-3-LG increased by 240.5%, 136.1%, and 66.3%, respectively, compared to that of the NCG. In addition, the CAT activities of SBP-III-3-HG, SBP-III-3-MG, and SBP-III-3-LGsignificantly increased by 159.5%, 87.9% and 74.7%, respectively, compared to that of the NCG (*p* < 0.05 or *p* < 0.01). These results suggested that SBP-III-3 could exert its anti-fatigue effects by enhancing the activities of antioxidant enzymes for eliminating the superfluous free radicals in organism.

Fatigue results in the release of ROS which cause lipid peroxidation of membrane structure. MDA, an oxidative degradation product of cell membrane lipids, is generally considered as an indicator of lipid peroxidation. In fatigue conditions, MDA level is increased and accompanied with a decrease in levels of the antioxidant enzymes GSH-Px and SOD [37]. Therefore, MDA is used as a biomarker to measure the level of oxidative stress in an organism. As shown in Table 2, the MDA levels in mice liver and plasma were decreased with the increase of SBP-III-3 dosage. In mice liver, the MDA levels of SBP-III-3-HG, SBP-III-3-MG, and SBP-III-3-LG significantly decreased by 53.6%, 49.9%, and 28.0%, respectively, compared to that of the NCG (*p* < 0.01). In addition, the MDA levels of SBP-III-3-HG, SBP-III-3-MG, and SBP-III-3-LG significantly lessen by 52.3%, 48.5%, and 41.0%, respectively, compared to that of the NCG in mice plasma (*p* < 0.01). The present data were in line with the decrease in levels of the antioxidant enzymes GSH-Px and SOD. These results indicated that SBP-III-3 might induce the MDA level by inhibiting lipid peroxidation of cell membrane lipids.

### 2.5. Amino Acid Composition of SBP-III-3

The bioactivities of protein hydrolysates and peptides were directly influenced by the amino acid compositions. Chen, Chi, Zhao, and Lv reported that Gly residue may contribute significantly to antioxidant activity since the single hydrogen atom in the side chain of Gly serves as a proton-donator and neutralizes active free radical species [38]. Nimalaratne, Bandara, and Wu also reported that the single hydrogen atom of Gly residue could provide a high flexibility to the peptide backbone and positively influence the antioxidant properties [39]. Pan et al. reported that hydrophobic amino acid residues including Ala, Leu, and Met played an important role in scavenging free radicals because the large hydrophobic group could help them make contact with hydrophobic radical species [10]. The pyrimidine ring of the Pro residue can increase the flexibility of the peptides and also be capable of quenching singlet oxygen due to its low ionization potential [38]. As shown in Table 2, the contents of Gly, Ala, and Pro residues in SBP-III-3 were 35.10%, 13.46%, and 11.28%, respectively, which reached to 59.84% of the total content of amino acids. In addition, the aromatic amino acid , such as Phe, Trp, His, and Tyr, with imidazole groups were also proved to have the ability to quench free radicals by direct electron transfer [11], and acid and basic amino acid residues, including Glu and Asp, were identified to have strong abilities to chelate metal ions, as well as scavenge HO•. Therefore, SBP-III-3 was rich in amino acids with antioxidant activity, which should be one of the important factors of its high antioxidant activity.

Amino acids were also found to play a key role in the regulatory metabolism involved in muscular activity. Vineyard et al. reported that feeding horses with an amino acid-based supplement every day might support muscle development during exercise and promote exercise metabolism and recovery [40]. The essential amino acid (EAA) ingestion during the exercise could attenuate the degradation of myofibrillar protein, thereby enhancing the exercise capability [41]. The EAA content of SBP-III-3 is 15.7%, which might be beneficial to its anti-fatigue capability. Glu residue was proved to have positive influence to the nervous system and would also be beneficial during exercise [42]. Marquezi et al. reported that Asp residue was advantageous in the oxidative deamination and could induce the blood ammonia concentration and postpone the occurrence of fatigue [43]. Therefore, SBP-III-3 contains 6.86% Glu residue and 4.54% Asp residue, suggesting that it might have a potential anti-fatigue effect. Furthermore, Bazzarre et al. reported that the content of amino acid residues, especially Gly, Ala, Val, Thr, Ile, Ser, and Tyr in the plasma will quickly reduce during an endurance test [44]. Table 3 showed that SBP-III-3 contained 57.72% of the above amino acid residues, which was higher than that of loach peptides (32.2%) with antioxidant activity and anti-fatigue effect [1]. The data indicated that these amino acids could enhance the exercise capability of SBP-III-3. In addition, the result of amino acid composition provided a basis for the good antioxidant and anti-fatigue capacities of SBP-III-3.

## 3. Experimental Section

### 3.1. Chemicals and Reagents

Frozen swim bladders of the croceine croaker (*P. crocea*) with an average body weight of 300–350 g were obtained from Zhejiang Dahaiyang Sci-Tech Co., Ltd. (Zhoushan, China). Alcalase, Sephadex G-25, and trifluoroacetic acid (TFA) were purchased from Ythx biotechnology Co., Ltd. (Beijing, China). All of the kits, including SOD, CAT, GSH-Px, MDA, BUN, lactic acid (LA), lactic dehydrogenase (LDH), liver glycogen, and muscle glycogen, were purchased from Nanjing Jiancheng Bioengineering Institute (Nanjing, China). Plasmid DNA (pBR322DNA) was purchased from TaKaRa Biotechnology Co., Ltd. (Dalian, China). Other reagents were of analytical grade and purchased from Sinopharm Chemical Reagent Co., Ltd. (Shanghai, China).

### 3.2. Preparation Protein Hydrolysate of Swim Bladders

Frozen swim bladders were unfrozen, cut into small pieces (0.5 × 0.5 cm) and soaked in 0.1 M NaOH with a solid/solvent ratio of 1:10 (*w*/*v*) to remove non-collagenous proteins. The mixture was continuously stirred for 24 h at 4 °C, and the NaOH solution was changed every 4 h. Thereafter, the residues were washed with cold distilled water to achieve the neutral pH. Washed samples were then suspended in 10% butyl alcohol for 12 h with a change of solution every 3 h. Defatted samples were thoroughly washed with cold water, homogenized, and suspended in phosphate buffer solution (PBS) with a solid/solvent of 1:3 and hydrolyzed for 8 h separately using alcalase at pH 9.0, 45 °C, papain at pH 7.0, 50 °C, pepsin at pH 3.0, 37 °C, and trypsin at pH 8.0, 60 °C with a total enzyme dose 2%. Enzymatic hydrolysis was stopped by heating for 10 min in boiling water, and hydrolysate was centrifuged at 8000× *g* for 10 min. The resulting supernatants using alcalase were lyophilized and named as SBP.

### 3.3. Isolation and Purification of Antioxidant Peptide from SBP

SBP was fractionated using ultrafiltration (8400, Millipore, Hangzhou, China) with MW cutoffs of 10 kDa, 5 kDa, and 3 kDa membranes, respectively, (Millipore, Hangzhou, China) for the lab scale at 0.30 MPa, 20 °C. Four fractions termed SBP-I (MW < 3 kDa), SBP-II (3 kDa < MW < 5 kDa), SBP-III (5 kDa < MW < 10 kDa), and SBP-IV (MW > 10 kDa) were pooled, concentrated, and lyophilized. SBP-III with the highest HO• scavenging activity among all ultrafiltration fractions was redissolved in distilled water and separated using a Sephadex G-25 gel filtration chromatography column (Φ2.6 cm × 60 cm, Huanyu Glass Co., Ltd., Xuchang, China) eluted with distilled water at a flow rate of 1.5 mL/min, and the eluate was monitored at 280 nm. Four peaks (SBP-III-1 to SBP-III-4) were collected and measured their HO• scavenging activities and SBP-III-3 was chosen for further analysis.

### 3.4. Degree of Hydrolysis (DH)

DH analysis was performed according to the previously described method [13]. The hydrolysate (50 μL) was mixed with 0.5 mL of 0.2 M phosphate buffer, pH 8.2, and 0.5 mL of 0.05% 2,4,6-trinitrobenzenesulfonic acid (TNBS) reagent. TNBS was freshly prepared before use by diluting with deionized water. The mixture was incubated at 50 °C for 1 h in a water bath. The reaction was stopped by adding 1 mL of 0.1 M HCl, incubating at room temperature for 30 min. The absorbance was monitored at 420 nm. L-leucine was used as a standard. To determine the total amino acid content, mungbean meal was completely hydrolysed with 6 M HCl with a sample to acid ratio of 1:100 at 120 °C for 24 h. DH (%) was calculated using the following equation:

DH = ((A_t_ − A_0_)/(A_max_ − A_0_)) × 100

where A_t_ was the amount of a-amino acids released at time t, A_0_ was the amount of a-amino acids in the supernatant at 0 h, and A_max_ was the total amount of a-amino acids obtained after acid hydrolysis at 120 °C for 24 h.

### 3.5. MW Distribution

MW distribution of SBP-III-1 to SBP-III-4 were determined by high-performance size exclusion chromatography (HPSEC) on a TSK-G3000SWXL column (TOSOH Corporation, Tokyo, Japan) using a high-pressure liquid chromatography system (Agilent 1200 HPLC, Agilent Ltd., Santa Rosa, CA, USA) [45]. The mobile phase consisted of 0.1 M sodium phosphate buffer (pH 7.0). A sample (20 μL) was eluted at a flow rate of 0.5 mL/min, and measured by monitoring the absorbance at 230 nm. The approximate MW was determined using standard protein samples (Sigma-Aldrich Co., LLC., St. Louis, MO, USA) as reference: thyroglobulin (670 kDa), γ-globulin (150 kDa), ovalbumin (44 kDa), trypsin inhibitor (20.1 kDa), ribonuclease A (14.7 kDa), Pro–Tyr–Phe–Asn–Lys (667 Da), and Trp–Asp–Arg (475 Da).

The calibration curve showed that the column separated the standard proteins well. The fitted linear equation between MW (logMW) and the retention time (R_t_, min) was calculated by the method of least squares, as Log MW = −0.2036R_t_ + 7.6164 (*R*^2^ = 0.9766). The MW of SBP-III-1 to SBP-III-4 was calculated by the elution time.

### 3.6. Amino Acid Composition Analysis

The amino acid composition of SBP-III-3 was analyzed according to the previous method [46]. To determine the amino acid composition, freeze-dried SBP-III-3 was dissolved in distilled water to obtain a concentration of 1 mg/mL, and an aliquot of 50 mL was dried and hydrolyzed in a vacuum-sealed glass tube at 110 °C for 24 h in the presence of 6 M HCl, which contained 0.1% phenol. Norleucine (Sigma Aldrich, Inc., St. Louis, MO, USA) was used as an internal standard. After hydrolysis, the samples were again vacuum-dried, dissolved in application buffer, and injected into an automated amino acid analyzer (HITACHI 835-50 Amino Acid Analyzer, Tokyo, Japan). Determination of tryptophan was also performed by HPLC analysis after alkaline hydrolysis [47]. Briefly, samples (5 mg) were dissolved in 3 mL of 4 N NaOH, sealed in hydrolysis tubes under nitrogen, and incubated in an oven at 100 °C for 4 h. Hydrolysates were cooled on ice, neutralized to pH 7 using 12 N HCl, and diluted to 25 mL with 1 M sodium borate buffer (pH 9). Aliquots of these solutions were filtered through a 0.45 μm Millex filter (Millipore, Hangzhou, China) prior to injection. Standard solutions of tryptophan were prepared by dilution of a stock solution (0.51 mg tryptophan/mL 4 N sodium hydroxide). They were diluted to 3 mL with 4 N sodium hydroxide and incubated as above. Finally, 20 μL samples and tryptophan solutions were determined by an HPLC system (Agilent 1260 HPLC, Agilent Ltd.).

### 3.7. Antioxidant Activity

#### 3.7.1. Radical Scavenging Activities

The DPPH•, HO•, $O_{2}^{-}$•, and ABTS^+^• scavenging activities were measured by the previous method [13], and the half elimination ratio (EC_50_) was defined as the concentration where a sample caused a 50% decrease of the initial concentration of DPPH•, HO•, $O_{2}^{-}$•, and ABTS^+^•, respectively.

##### HO• Scavenging Activity

First, 1.0 mL of a 1.865 mM 1,10-phenanthroline solution and 2.0 mL of the sample were added to a screw-capped tube and mixed. Then, 1.0 mL of a FeSO_4_·7H_2_O solution (1.865 mM) was added to the mixture. The reaction was initiated by adding 1.0 mL of H_2_O_2_ (0.03%, *v*/*v*). After incubating at 37 °C for 60 min in a water bath, the absorbance of the reaction mixture was measured at 536 nm against a reagent blank. The reaction mixture without any antioxidant was used as the negative control, and a mixture without H_2_O_2_ was used as the blank. The HO• scavenging activity was calculated using the following formula:

HO• scavenging activity (%) = ((A_s_ − A_n_)/(A_b_ − A_n_)) × 100

where A_s_, A_n_, and A_b_ are the absorbance values determined at 536 nm of the sample, the negative control, and the blank after the reaction, respectively.

##### DPPH• Scavenging Activity

Two millilitres of samples consisting of distilled water and different concentrations of the analytes were placed in cuvettes, and 500 μL of an ethanolic solution of DPPH (0.02%) and 1.0 mL of ethanol were added. A control sample containing the DPPH solution without the sample was also prepared. In the blank, the DPPH solution was substituted with ethanol. The antioxidant activity of the sample was evaluated using the inhibition percentage of the DPPH• with the following equation:

DPPH• scavenging activity (%) = (A_0_ + A′ − A)/A_0_ × 100

where A is the absorbance rate of the sample, A_0_ is the control group absorbance, and A′ is the blank absorbance.

##### $O_{2}^{-}$• Scavenging Activity

In the experiment, superoxide anions were generated in 1 mL of nitrotetrazolium blue chloride (NBT) (2.52 mM), 1 mL of NADH (624 mM), and 1 mL of different sample concentrations. The reaction was initiated by adding 1 mL of phenazinemethosulphate (PMS) solution (120 μM) to the reaction mixture. The absorbance was measured at 560 nm against the corresponding blank after 5-min incubation at 25 °C. The scavenging capacity of the $O_{2}^{-}$• was calculated using the following equation:

$$O_{2}^{-}•\text{ scavenging activity }(\%)=((A_{\mathrm{control}}-A_{\mathrm{sample}})/A_{\mathrm{control}})\times 100\%,$$

where A_control_ is the absorbance without the sample and A_sample_ is the absorbance with the sample.

##### ABTS^+^• Scavenging Activity

ABTS^+^• was generated by mixing an ABTS stock solution (7 mM) with potassium persulphate (2.45 mM). The mixture was left in the dark for 16 h at room temperature. The ABTS^+^• solution was diluted in 5 mM phosphate-buffered saline (PBS, pH 7.4) to an absorbance of 0.70 ± 0.02 at 734 nm. One millilitre of diluted ABTS^+^• solution was mixed with one millilitre of the different sample concentrations. Ten minutes later, the absorbances were measured at 734 nm against the corresponding blank. The ABTS^+^• scavenging activities of the samples were calculated using the same equation as indicated in $O_{2}^{-}$• scavenging activity (%).

#### 3.7.2. DNA Damage Protective Effect

The ability of SBP-III-3 to protect supercoiled pBR322 plasmid DNA was measured by the previous method with a slight modification [28]. The reaction mixtures (15 μL) contained 5 μL of PBS (10 mM, pH 7.4), 1 μL of plasmid DNA (0.5 μg), 5 μL of the SBP-III-3, 2 μL of 1 mM FeSO_4_, and 2 μL of 1 mM H_2_O_2_ were incubated at 37 °C for 30 min. After incubation, 2 μL of a loading buffer (50% glycerol (*v*/*v*), 40 mM EDTA and 0.05% bromophenol blue) was added to stop the reaction and the reaction mixtures were electrophoresed on 1% agarose gel containing 0.5 μg/mL ethidium bromide in Tris/acetate/EDTA gel buffer for 50 min (60 V), and the DNA in the gel was visualized and photographed under ultraviolet light. Ascorbic acid was used as a positive control.

### 3.8. Animals and Experimental Diets

Male Institute of Cancer Research (ICR) mice with an average body weight of 20–25 g were purchased from the Zhejiang Academy of Medical Sciences (China). All of the in vivo tests were carried by the School of Food and Pharmacy of Zhejiang Ocean University (China), which obtained the permission for performing the research protocols and all animal experiments conducted during the present study from the ethics committee of Zhejiang Ocean University. All experimental procedures were conducted under the oversight and approval of the Academy of Experimental Animal Center of Zhejiang Ocean University and in strict accordance with the NIH Guide for the Care and Use of Laboratory Animals (NIH, 2002).

In vivo anti-fatigue activity was determined on the previous method with a slight modification [3,48]. Male ICR mice were housed in a SPF level laboratory under controlled temperature of 21 ± 1 °C with moderate humidity of 55% ± 5%, and air flow conditions in a 12 h light/dark cycle; noise was <60 dB. Mice had free access to the standard diet and water during the experiments. After one week adaptation, 48 mice were randomly divided into four groups (12 mice per group): the normal control group (NCG) and three swim bladder peptide (SBP-III-3) treatment groups. Mice in NCG were administered with physiological saline (0.1 mL/10 g body weight per day) by gastrogavage; mice in SBP-III-3 treatment groups were respectively fed with SBP-III-3 in three different doses (50, 100, and 200 mg/kg body weight per day) by gastrogavage, and the three groups were accordingly named as low-dose group (SBP-III-3-LG), middle-dose group (SBP-III-3-MG), and high-dose group (SBP-III-3-HG).

### 3.9. In Vivo Anti-Fatigue Effect of SBP-III-3

#### 3.9.1. Weight-Loaded Swimming Test in ICR Mice

Weight-loaded swimming test was according to the previous method with some modifications [49,50]. Physiological saline/SBP-III-3 were administrated orally (8:00 a.m.) to mice of the normal control group (NCG) and three swim bladder peptide (SBP-III-3) treatment groups (SBP-III-3-LG, SBP-III-3-MG, and SBP-III-3-HG) once daily for 28 days. After the treatment with SBP-III-3 or physiological saline for 30 min at the last time point, the loaded swimming experiment was carried out. All of the mice were weighed and loaded with an iron ring equaling 5% of each mouse’s body weight on the tail root of each mouse; they were placed in a swimming pool (50 cm × 50 cm × 40 cm) with 30 cm deep water with 25 ± 1°C. The swimming time of mice was calculated from the time they began to swim up to the time the exhibited exhaustion, which was determined as a loss of coordinated movements and failure to return to the surface within 10 s. The length of the swimming time to exhaustion was evaluated as the degree of fatigue.

#### 3.9.2. Biochemical Parameter Determination on Anti-Fatigue

Biochemical parameters were determined according to the method described by You et al. [1], and were performed according to the recommended procedures provided by the kits (Nanjing Jiancheng Bioengineering Institute, Nanjing, China). Briefly, two days later after weight-loaded swimming test, all the mice were forced to swim for 90 min without a load after gavage for 30 min, then anesthetized with 10% chloral hydrate after 60 min rest, and the mice were sacrificed by cervical dislocation.

For the serum assays, 250 L of arterial blood was respectively collected from left femoral artery to determine MDA, BUN, and LA content and LDH, SOD, CAT, and GSH-Px activity using commercial diagnostic kits. The GSH-Px has the ability to decompose hydrogen peroxide (H_2_O_2_) and other organic hydroperoxides (ROOH). The reaction uses GSH to complete the reaction using H_2_O_2_, as the substrate. The consumption of nicotinamide adenine dinucleotide phosphate (NADPH) is used to determine the GSH-Px activity. The catalase activity was determined colorimetrically with a CAT assay kit, which is based on the decomposition of the H_2_O_2_ optical density at 415 nm by CAT.

For the hepatic and muscular assays, the muscles and livers of the mice were also taken to determine the content of MDA and glycogen. The muscles and livers of the mice were dissected immediately after removal, washed with 0.9% saline, and blotted dry with filter paper. Liver samples (∼100 mg) were accurately weighed, and homogenized in 8 mL of homogenization buffer. The content of MDA and glycogen was determined according to the recommended procedures.

### 3.10. Statistical Analysis

The data are reported as the mean ± standard deviation (SD). An ANOVA test using SPSS 19.0 (Statistical Program for Social Sciences, SPSS Corporation, Chicago, IL, USA) was used to analyze the experimental data. Duncan’s multiple range test was used to measure the differences among the parameters means. The differences were considered significant if *p* < 0.05 or *p* < 0.01.

## 4. Conclusions

Our study firstly prepared and evaluated the high antioxidant and anti-fatigue activities of the peptide fraction (SBP-III-3) from the croceine croaker (*P. crocea*) swim bladder. The results showed that SBP-III-3 could effectively scavenge HO•, DPPH•, $O_{2}^{-}$•, and ABTS^+^•, prolong the swimming time to exhaustion of mice, decreased the BUN, LA, and MDA levels, and increase the LDH, liver glycogen, and muscle glycogen levels in mice. In addition, SBP-III-3 could improve the activities of SOD, GSH-Px, and CAT in vivo. These results confirmed that SBP-III-3 possessed good antioxidant and anti-fatigue capacities in mice and provided an important basis for developing the swim bladder peptide served as a novel functional food.

Protein hydrolysates are composed of free amino acids and short-chain peptides that exhibit numerous advantages as nutraceuticals, functional foods, or medicines. Structure-activity studies of antioxidant peptides reported that peptides and protein hydrolysates display different activities depending on the peptide size, the amino acid sequence, and the presence of amino acids involved in oxidative reactions. Therefore, further research should be done in order to purify and identify antioxidant and anti-fatigue peptides from SBP-III-3, and more detailed studies on physiological functions, pharmacological effects, and structure-activity relationship of SBP-III-3 and the purified peptides will also be needed.

## Figures and Tables

**Table 1:** HO• scavenging activities of protein hydrolysate of scalloped hammerhead cartilage using different proteases (*c* = 15 mg protein/mL). All of the values were mean ± standard deviation (SD) (*n* = 3).

Protease	Enzymolysis Condition	Degree of Hydrolysis (DH%)	HO• Scavenging Rate (%)
**Papain**	pH 7.0, 60 °C, 4 h, total enzyme dose 2.5%	17.84 ± 0.71 ^a^	34.85 ± 1.05 ^a^
**Alcalase**	pH 8.0, 50 °C, 4 h, total enzyme dose 2.5%	22.32 ± 0.74 ^b^	54.76 ± 1.94 ^b^
**Trypsin**	pH 8.0, 40 °C, 4 h, total enzyme dose 2.5%	16.21 ± 0.37 ^c^	62.38 ± 1.67 ^c^
**Pepsin**	pH 2.0, 37 °C, 4 h, total enzyme dose 2.5%	19.52 ± 0.49 ^d^	55.47 ± 2.02 ^b^
**Neutrase**	pH 6.0, 50 °C, 4 h, total enzyme dose 2.5%	21.37 ± 0.67 ^b^	50.67 ± 1.85 ^d^

^a–d^ Values with different letters indicated significant differences at the same concentration (*p* < 0.05).

**Table 2:** Effects of SBP-III-3 on BUN, LA, LDH, liver glycogen, muscle glycogen, SOD, GSH-Px, CAT, and MDA in mice (*n* = 3).

	NCG	SBP-III-3-LG	SBP-III-3-MG	SBP-III-3-HG
BUN (mmol/L)	9.34 ± 0.39	7.81 ± 0.61 **	7.79 ± 0.47 **	7.28 ± 0.43 **^,a^
LA (mmol/L)	3.08 ± 0.21	2.65 ± 0.47 *	2.56 ± 0.35 *	2.46 ± 0.34 *
LDH (U/gprot)	2784.95 ± 322.92	3397.10 ± 215.90	3605.87 ± 315.21 *	3690.76 ± 337.18 *
Liver glycogen (mg/g)	8.32 ± 0.47	11.29 ± 2.31 *	17.36 ± 1.16 **	21.41 ± 5.23 **^,b^
Muscle glycogen (mg/g)	2.23 ± 0.56	2.44 ± 0.36	3.39 ± 0.35 *	4.80 ± 1.12 *
SOD (U/mg prot)	68.82 ± 6.17	71.74 ± 2.52	79.63 ± 7.40 *	99.24 ± 4.38 **^,b^
GSH-Px (IU)	43.22 ± 4.09	71.89 ± 2.34	102.05 ± 5.78 **	147.16 ± 12.80 **^,b^
CAT (U/g prot)	186.14 ± 2.26	325.27 ± 1.52 *	349.75 ± 4.09 *	483.00 ± 5.87 **^,a^
MDA in liver (mmol/L)	2.39 ± 0.55	1.72 ± 0.25 **	1.23 ± 0.31 **^,a^	1.11 ± 0.23 **^,b^
MDA in plasma (mmol/L)	19.92 ± 2.87	11.75 ± 2.62 **	9.97 ± 1.31 **	9.50 ± 0.55 **

* *p* < 0.05, ** *p* < 0.01 compared with the control; ^a^ *p* < 0.05, ^b^ *p* < 0.01 compared with the low group.

**Table 3:** The amino acid composition of SBP-III-3 (*n* = 3).

Amino Acid	Concentration (μmol/L)	Composition (%)
Asp	871.33 ± 18.56	4.60 ± 0.10
Glu	1317.30 ± 35.14	6.86 ± 0.18
Ser	540.61 ± 20.47	2.82 ± 0.11
Gly	6744.51 ± 143.47	35.10 ± 0.75
His	86.39 ± 2.34	0.45 ± 0.04
Arg	718.84 ± 12.38	3.74 ± 0.06
Thr	450.29 ± 13.54	2.34 ± 0.07
Ala	2343.97 ± 34.56	13.46 ± 0.19
Pro	2165.95 ± 40.68	11.28 ± 0.21
Hyp	1629.48 ± 26.87	8.49 ± 0.14
Tyr	60.96 ± 1.35	0.32 ± 0.01
Val	499.64 ± 12.58	2.60 ± 0.07
Met	244.28 ± 8.51	1.27 ± 0.05
Ile	207.49 ± 8.34	1.08 ± 0.04
Leu	388.61 ± 12.97	2.02 ± 0.07
Phe	233.10 ± 9.08	1.21 ± 0.05
Lys	453.21 ± 14.58	2.36 ± 0.08
Essential amino acid (EAA)	3017.23 ± 86.37	15.70 ± 0.45
Total	18,955.96 ± 315.20	100%
